# Congratulations to Prof. em. Garth L. Nicolson – co-founder of Clinical & Experimental Metastasis

**DOI:** 10.1007/s10585-023-10239-1

**Published:** 2023-10-21

**Authors:** Jörg Haier, Jonathan Sleeman

**Affiliations:** 1 Comprehensive Cancer Center Hannover, Hannover Medical School, Hannover, Germany; 2 Medical Faculty Mannheim, European Center for Angioscience, University of Heidelberg, Ludolf-Krehl-Str. 13 - 17, D-68167 Mannheim, Germany; 3 Karlsruher Institut f?r Technologie (KIT) IBCS-BIP, Hermann-von-Helmholtz-Platz 1, 76344 Eggenstein-Leopoldshafen, Germany

Professor Emeritus Garth L. Nicolson, one of the three founder editors of Clinical & Experimental Metastasis, recently celebrated his 80th birthday. On behalf of the Editorial Board, we warmly congratulate him and want to take this opportunity to thank him for his lifetime efforts and contributions to both our journal and the Metastasis Research Society (MRS).

In addition to being co-founder of Clinical & Experimental Metastasis together with Kurt Hellmann and Tatsuro Irimura, Garth Nicolson also served as President of the MRS (1988–1990) and contributed to the success of the MRS through taking on numerous additional functions within the society. Through these activities he paved the way for today’s vision of building connections between researchers and fostering the next generation of emerging leaders. He made important early contributions to metastasis research at a time when metastasis was considered difficult to address experimentally, laying the foundation for the current progressive expansion of research efforts that are dedicated to understanding, preventing and treating advanced cancer.

Garth Nicolson graduated from the University of California, Los Angeles, with a major in chemistry in 1965 and carried out research in the field of biochemistry at the University of California, San Diego, from where he earned his PhD in 1970. As a biochemist he developed the landmark scientific model for cell membranes, known as the Fluid Mosaic Model.^1^ In subsequent years, he became research and postdoctoral fellow at various prestigious institutes, including the Salk Institute, where he developed interests that led to a scientific career dedicated to cancer research. After taking up various other positions, Garth Nicolson became the David Bruton Jr. Chair in Cancer Research and Professor and Chairman at the University of Texas M.D. Anderson Cancer Center in Houston. He was also Professor of Internal Medicine and Professor of Pathology and Laboratory Medicine at the University of Texas Medical School, Houston. Later he became Founder, President, Chief Scientific Officer and Emeritus Research Professor of Molecular Pathology at the Institute for Molecular Medicine in Huntington Beach, California. Furthermore, he is a Conjoint Emeritus Professor at the University of Newcastle (Australia).

In recognition of the importance of his work, Garth Nicolson has won many awards, including the Burroughs Wellcome Medal of the Royal Society of Medicine (United Kingdom), the Stephen Paget Award of the Metastasis Research Society, the National Cancer Institute Outstanding Investigator Award, the Innovative Medicine Award of Canada and the EU Academy of Sciences. Professor Nicolson has published the results of his research work in over 700 medical and scientific papers, and has edited 20 books. He has served on the Editorial Boards of 30 medical and scientific journals and was Senior Editor of four of these, one of which was Clinical & Experimental Metastasis.

In addition to his own scientific engagement and success, Garth Nicolson has educated a large number of research fellows during his long and successful scientific career. Many of his trainees have subsequently made seminal contributions to metastasis research and other cancer-related research fields during their own scientific careers, underlining his continued impact and legacy through those he has mentored and inspired. With much gratitude, we wish him all the very best as he enters his 9th decade of life.

## Footnotes

1 Singer, S. J.; Nicolson, G. L. (1972). “The Fluid Mosaic Model of the Structure of Cell Membranes”. Science. 175 (4023): 720–731.

